# DNA Methylome Changes of Muscle- and Neuronal-Related Processes Precede Bladder Cancer Invasiveness

**DOI:** 10.3390/cancers14030487

**Published:** 2022-01-19

**Authors:** Maria Bošković, Blanka Roje, Felicia Fei-Lei Chung, Andrea Gelemanović, Vincent Cahais, Cyrille Cuenin, Rita Khoueiry, Katarina Vilović, Zdenko Herceg, Janoš Terzić

**Affiliations:** 1 Laboratory for Cancer Research, University of Split School of Medicine, Šoltanska 2, 21000 Split, Croatia; maria.boskovic@mefst.hr (M.B.); blanka.roje@mefst.hr (B.R.); katarina.vilovic@mefst.hr (K.V.); 2 Epigenomics and Mechanisms Branch, International Agency for Research on Cancer (IARC), 150 Cours Albert Thomas, 69008 Lyon, France; feliciacfl@sunway.edu.my (F.F.-L.C.); CahaisV@iarc.fr (V.C.); cueninc@iarc.fr (C.C.); KhoueiryR@iarc.fr (R.K.); 3 Department of Medical Sciences, School of Medical and Life Sciences, Sunway University, Jalan Universiti, Bandar Sunway, Petaling Jaya 47500, Selangor, Malaysia; 4 Biology of Robustness Group, Mediterranean Institute for Life Sciences (MedILS), Šetalište Ivana Meštrovića 45, 21000 Split, Croatia; andrea.gelemanovic@gmail.com

**Keywords:** bladder cancer, DNA methylation, gene expression, invasiveness, muscle contraction, neuronal system

## Abstract

**Simple Summary:**

Urinary bladder cancer can be therapeutically controlled until it becomes invasive, thus identifying critical molecular processes preceding and promoting the transition from pre-invasive to invasive tumors is of vital medical importance. Here, we tested epigenomic (DNA methylation) and gene expression profiles in non-invasive and invasive bladder cancers. We found methylation changes in the genes related to muscle and neuronal processes that discriminate between two cancer stages. Our results may open new avenues for early diagnosis of pre-invasive tumors by testing methylation profiles of tumor cells present in patients’ urine or biopsies leading to timely therapeutic measures.

**Abstract:**

Bladder cancer (BC) is the ninth leading cause of cancer death with one of the highest recurrence rates among all cancers. One of the main risks for BC development is exposure to nitrosamines present in tobacco smoke or in other products. Aberrant epigenetic (DNA methylation) changes accompanied by deregulated gene expression are an important element of cancer pathogenesis. Therefore, we aimed to determine DNA methylation signatures and their impacts on gene expression in mice treated with N-butyl-N-(4-hydroxybutyl) nitrosamine (BBN), a carcinogen similar to compounds found in tobacco smoke. Following BBN administration mice developed non-invasive or invasive bladder cancers. Surprisingly, muscle- and neuronal-related pathways emerged as the most affected in those tumors. Hypo- and hypermethylation changes were present within non-invasive BC, across CpGs mapping to the genes involved in muscle- and neuronal-related pathways, however, methylation differences were not sufficient to affect the expression of the majority of associated genes. Conversely, invasive tumors displayed hypermethylation changes that were linked with alterations in gene expression profiles. Together, these findings indicate that bladder cancer progression could be revealed through methylation profiling at the pre-invasive cancer stage that could assist monitoring of cancer patients and guide novel therapeutic approaches.

## 1. Introduction

Bladder cancer (BC) is the ninth most frequently diagnosed cancer worldwide, as well as the most common urogenital carcinoma, with a three-times-higher rate of occurrence among men than women [1,2]. While the majority of BC patients display a non-muscle-invasive form of BC, an important fraction is diagnosed with a muscle-invasive form of malignancy [3]. These distinctions should be taken into consideration for the treatment and clinical outcome of BCs [4], although the molecular mechanisms underpinning these differences are yet to be elucidated. Well-known risk factors for BC development include the consumption of polluted water or food (e.g., arsenic contamination), infection with *Schistosoma haematobium*, occupational exposures to aromatic amines and, the leading risk factor for BC, tobacco smoking [5]. Approximately half of all BC cases have been attributed to tobacco smoking, with a lag time of 20 to 30 years between exposure and diagnosis [1,6].

It is well established that epigenetic mechanisms may act as “sensors” of adverse exposure and “mediators” of the cell response to endogenous cues and environmental stressors [7,8]. Consistent with this notion, disruption of epigenome regulation and the resulting gene expression alterations is a major mechanism underlying tumor development and progression [9]. Major sequencing efforts revealed that virtually all cancer types harbor numerous changes in the epigenome (notably in DNA methylome) [10,11] and that these changes may be risk factor specific (signatures) [7]. Moreover, recent studies suggested that epigenetic deregulation may be an early event in tumorigenic processes [12,13]. However, the observed changes in epigenome profiles in tumor tissues represent snapshot portraits of accumulated epigenetic events captured at a given (often advanced) time point of a multistep process [11]; thus, the critical epigenetic events that precede and promote the development and progression of different cancer types are poorly characterized.

Several previous studies investigated epigenetic (DNA methylation) analysis in bladder tumors using candidate genes or genome-wide approaches [14,15]. However, these studies were often limited by epigenome coverage, and the epigenetic changes in tumor stages were not considered.

For studying BC in rodents, the most commonly used carcinogen is N-butyl-N-(4-hydroxybutyl) nitrosamine (BBN), which has a selective mechanism of action directed to the urinary bladder [16]. BBN is structurally related to chemical carcinogens found in tobacco smoke [17,18]. Furthermore, BBN-induced bladder tumors in mice have histological and mutational similarities with human tumors [19] therefore the BBN mouse-model offers a convenient approach for investigating molecular underpinnings of BC development.

An increasing amount of evidence show that aberrant DNA methylation has implications in cancer development [10,20]. In line with that, it’s been demonstrated that aberrant DNA methylation, followed by chromatin remodeling, could be a driver event in BC pathogenesis [9,21]. However, many aspects of the role of DNA methylation changes in BC development and phenotypes (invasive and non-invasive) are poorly investigated. Here, we aimed to characterize histological and DNA methylation changes occurring in the BBN-mouse model with its reflection on the gene expression. We showed that methylation changes could be detected in the non-invasive BC in muscle- and neuronal-related processes before changes in gene expression were detectable, while in the invasive tumors they represent the most transcriptionally repressed processes. Since BC has one of the highest lifetime treatment costs per patient, and one of the highest recurrence rates among all cancers, the determination of epigenetic signatures of this malignancy could contribute to defining prognostic and diagnostic biomarkers for BC management [19]. Epigenetic signatures in BC could represent a powerful approach for monitoring the disease, as well as for the development of a new epigenetic-directed therapeutic strategies, which could be combined with current therapeutic options.

## 2. Materials and Methods

### 2.1. Animal Work

C57BL6/J wild type mice were purchased from the Jackson Laboratory. Animals were maintained in standard husbandry conditions with a 12 h light/dark cycle with a controlled temperature (21–24 °C) and humidity, while food and water (autoclaved) were available *ad libitum*. Animal experimental procedures were approved by the local institutional animal care and use committee (IACUC) and the Ministry of Agriculture of the Republic of Croatia (permit numbers: 525-10/0255-14-4 and 525-10/0543-20-3).

### 2.2. BBN-Induced Mouse Model of BC

**Figure 1:**
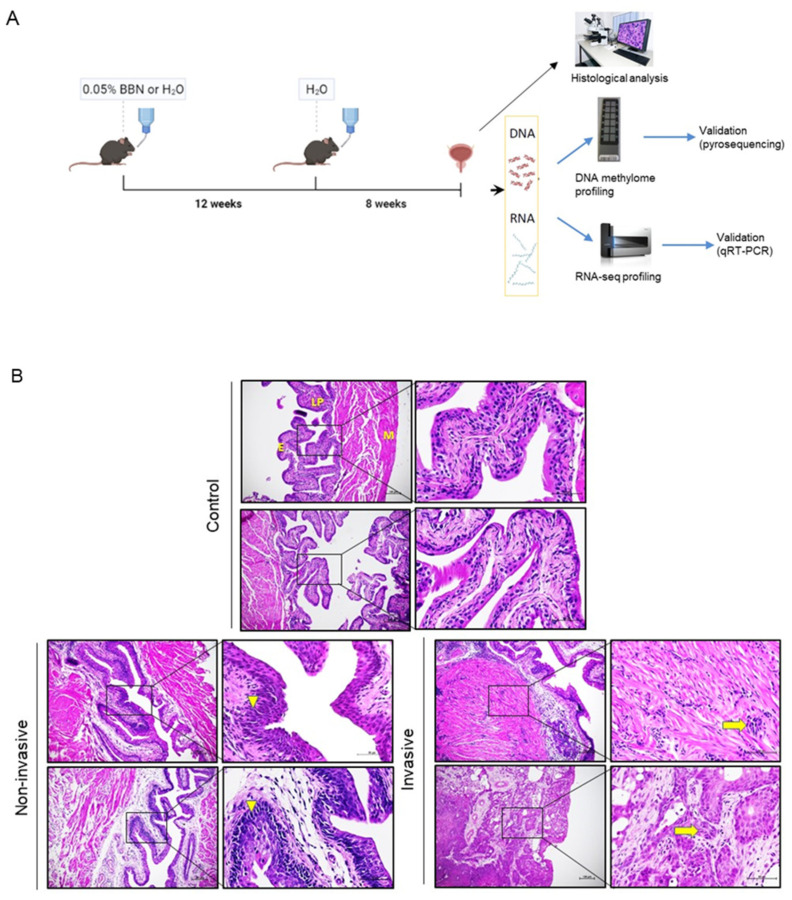
BBN treatment and bladder cancer morphology. (**A**) A schematic representation of BBN treatment (created with BioRender.com) and flow through experiments. (**B**) Representative images of patho-histological classifications of hematoxylin–eosin-stained murine bladders of control group (E, epithelium; LP, lamina propria; M, muscle) and after BBN exposure; non-invasive tumors carcinoma in situ (arrowheads indicate randomly arranged cells and compact, large, pleomorphic nuclei with multiple nuclei) and invasive (arrows point to urothelial cells invading the muscle) tumors. Images were taken at 400× and 600× magnifications.

Detailed description of the protocol can be found as described [22]. Briefly, two-month-old male mice were randomly assigned to the BBN-treated group (chronic treatment, *n* = 17) or untreated control group (*n* = 11) (Figure 1A). BBN-treated mice were given autoclaved tap water containing 0.05% BBN (*v*/*v*) (TCI Europe, Zwijndrecht, Belgium). Chronic BBN exposure consisted of administering BBN in the drinking water for 12 weeks followed by administering water without BBN for 8 weeks (complete duration was 20 weeks). BBN-treated mice were paired with age-adjusted controls, which were given autoclaved tap water for the period of BBN treatment for 20 weeks (*n* = 11). BBN-treated mice and controls were sacrificed by cervical dislocation at the end of the treatment. Urinary bladders were collected immediately following sacrifice and cut medially by a histological knife into approximately two halves. Half was snap-frozen for molecular analyses and the other half was immersed in 4% paraformaldehyde (PFA) (Sigma-Aldrich, Darmstadt, Germany) in PBS of pH 7.4 for paraffin-embedding and histological analysis. Frozen tissue was homogenized; part of ground tissue was used for DNA isolation and part for RNA extraction.

### 2.3. Histological Analysis

Following overnight fixation in 4% PFA, tissues were dehydrated using a series of ethanol dilutions (one hour in 75%, 90%, 95% and three times 100% ethanol), then cleared of ethanol with xylene (three times for 30 min), and embedded in paraffin (2 series of immersion in paraffin for 1 h and embedding in third paraffin). Sections for histological analysis were prepared from the paraffin-embedded tissue in 4–5-µm-thick sections with microtome (RM2125 RTS, Leica, Buffalo Grove, IL, USA) and stained with hematoxylin (Sigma-Aldrich, Darmstadt, Germany) and eosin (Merck, Darmstadt, Germany). Microscopical examination was done by researchers and a trained pathologist in a blinded manner. Images were captured using an Olympus BX43 microscope (Olympus Corporation, Tokyo, Japan).

### 2.4. DNA Extraction and Mouse Methylation BeadChip Array

DNA was extracted from bladder tissues using a DNeasy Blood and Tissue Kit (Qiagen, Hilden, Germany), according to the manufacturer’s instructions. All DNA samples were quantified by Qubit and 500 ng of DNA was bisulfite converted using an EZ DNA Methylation kit (Zymo Research, Irvine, CA, USA). Input of 250 ng bisulfite-converted DNA was used on an Infinium Mouse Methylation BeadChip (Illumina Inc., San Diego, CA, USA) which simultaneously interrogated more than 285,000 CpG sites across the murine genome at single-nucleotide resolution. For the Mouse Methylation BeadChip Array we used DNA obtained from two bladders with invasive tumors, three bladders with non-invasive tumors and three non-treated control bladders (the same samples were used for RNA sequencing).

### 2.5. Differential DNA Methylation Analysis

Raw data files were pre-processed, “Noob” normalization performed and quality control conducted using the SeSAMe package [23]. All samples clustered with high signal intensity on both green and red channels (Supplementary Figure S1A) and exhibited consistent raw signal intensities in both the green and red channels (Supplementary Figure S1B). Betas were normalized with the Noob method; and low-quality probes defined as probes exhibiting detection *p*-values > 0.02 and probes that exhibited NA values in more than 20% of the samples were removed from the analysis. The remaining missing values were replaced by group mean betas. Beta values were converted to M-values using the beta2m R function as implemented in lumi [24]. Intergroup comparisons were conducted using linear regression analysis with the limma R package [25] while controlling for multiple testing. This was followed by differentially methylated region (DMR) analysis on the significantly differentially methylated probes (DMPs, FDR < 0.05) using the DMRcate package [26].

### 2.6. Total RNA Extraction and Sample Quality Control

Total RNA was extracted using Minilys homogenizer (Bertin, Montigny-le-Bretonneux, France) with TRIzol reagent following the manufacturer’s instruction (Thermo Fisher Scientific, Waltham, MA, USA). Agarose gel electrophoresis was performed to check genomic DNA contaminations, which were not detected. Quantities and purities of the extracted RNA were assessed by NanoDrop.

### 2.7. RNA Sequencing

RNA sequencing libraries were prepared in Novogene (Cambridge, UK) consisted of mRNA purification (400 ng) using poly-T oligo-attached magnetic beads, cDNA preparation followed by library construction with end repair, A-tailing, adapter ligation, size selection, amplification, and purification. For RNA-seq we used RNA extracted from two bladders with invasive tumors, three bladders with non-invasive tumors and three non-treated control bladders. RNA was quantified using a Qubit and real-time PCR, while Bioanalyzer (Agilent Technologies, Inc., Santa Clara, CA, USA) was used for size distribution detection, sample purity, and integrity. Next-generation sequencing was done in the Novogene facility using an Illumina HWI-ST1276 machine. The average size of fragments ws 150 bp and pair-end sequencing was performed. The base number of raw data was at least 12 G of data per sample with 40 million paired reads in total.

### 2.8. Differential Gene Expression Analysis

Raw RNA-seq reads were aligned to the mouse reference genome (assembly GRCm39 GCA_000001635_8) and quantification was done using the FeatureCounts algorithm v1.5.0-p3. Differential gene expression was analyzed using the DESeq2 package v1.340 [27]. Raw counts were first pre-filtered to remove genes with less than 10 reads in total, normalized with DESeq2 default parameters, and transformed using variance stabilizing transformation (VST) to examine sample clustering via principal component analysis (PCA) and heatmap. For effect size shrinkage the apeglm method was used. Differentially expressed genes (DEGs) were defined with false discovery rate (FDR) -adjusted *p*-value < 0.05 and |log_2_ fold change| > 1.

### 2.9. Weighted Gene Co-Expression Network Analysis (WGCNA)

To assess the correlation patterns among the genes based on their transcriptome profiles and to perform clustering of highly correlated genes, weighted gene co-expression network analysis (WGCNA) was applied using the WGCNA package v1.70-3 [28,29]. To obtain a fully unsupervised correlation network, all genes were used as an input as VST-transformed counts. First, soft-thresholding power β was set to nine to achieve approximate scale-free topology, which is a prerequisite in weighted gene networks. To construct the network and detect gene modules, default WGCNA parameters were used, except that minimal module size was set to 1000 and the signed network option was chosen. Modules of highly correlated genes were then correlated with phenotype, and, for the significantly correlated modules (identified with *p*-value < 0.05), gene significance and module membership were calculated to quantify the associations of individual genes with the phenotype. Those genes that showed significant and high gene significance (|GS| > 0.8) and module membership (|MM| > 0.8) were selected as central module genes. Finally, one top hub gene was selected per module.

### 2.10. Functional Enrichment Analysis

Gene names were converted into Entrez IDs using the BiomaRt package v2.50.1 [30,31] with the Ensembl database. Functional enrichment analyses were performed with clusterProfiler package v3.16.1 [32,33] using Reactome Pathways from ReactomePA package v1.32.0 [34]. Over-representation tests were performed separately for significantly hypo- and hypermethylated DMRs, significantly down- and up-regulated DEGs, significantly correlated modules using only central module genes and for overlap between significant DMRs and DEGs. Statistically significantly enriched pathways were defined with FDR adjusted *p*-value < 0.05.

### 2.11. Pyrosequencing

DNA was quantified by Qubit followed by bisulfite conversion of 500 ng of DNA with an EZ DNA Methylation kit (Zymo Research, Irvine, CA, USA) according to the manufacturer’s protocol. Genomic regions of selected genes were PCR-amplified with previously established PCR conditions (see Supplementary Table S1). Then, briefly, the PCR product was bound to streptavidin sepharose beads (Cytiva, Uppsala, Sweden) followed by washing in 70% ethanol, denaturation in 0.2 M NaOH solution and washing in 1M Tris-HCl pH 7.6. After that, 0.4 μM of sequencing primer was annealed to the purified single-stranded PCR product and pyrosequencing was done using a PyroMark Gold reagent kit on the PyroMark Q96ID instrument (Qiagen, Hilden, Germany). For this purpose, we used DNA isolated from non-treated controls (*n* = 8), BBN-treated with invasive (*n* = 9) and non-invasive (*n* = 3) tumors. To quantify the methylation level at individual CpG dinucleotides, each locus was calculated as the percentage of methylated cytosines over all cytosines. The average was used in statistical analysis. Two-way ANOVA and Kruskal–Wallis tests were used to determine significance, *p*-values < 0.05 were considered significant. Normality and lognormality of data was determined by the Shapiro–Wilk test.

### 2.12. cDNA Preparation and qPCR

Reverse transcription was carried out with 1 µg of total mRNA using a High-Capacity cDNA Reverse Transcription Kit according to the manufacturer’s instructions (Applied Biosystems, Waltham, MA, USA) and analyzed by qPCR using SYBR Green MasterMix (BioRad, Hercules, CA, USA) on a BioRad CFX96 Real-Time System (C1000 Touch Thermal Cycle). Here we used RNA isolated from controls (*n* = 5), BBN-treated with invasive (*n* = 6) and non-invasive (*n* = 2) tumor phenotypes. Gene expression was normalized to actin beta (which was found to be stably expressed in RNA-seq analysis) and analyzed using the comparative Ct method. Primer sequences can be found in Supplementary Table S2. Unpaired T-tests and Mann–Whitney tests were used to determine significance between controls and invasions, while the non-invasive group was not involved in the statistics because the number of tested samples in the group was two. *p*-values < 0.05 were considered significant. Normality and lognormality of data were determined by the Shapiro–Wilk test.

### 2.13. Statistical Analysis and Figure Preparation

The complete bioinformatic pipeline was done in R v4.0.0 [35]. Principal component analyses (PCA) were performed using the base R function prcomp. Heatmaps were generated with the *Complex Heatmap* package v2.4.3 [34]. Venn diagrams were created with the eulerr package v6.1.1. Reactome pathways were visualized as dotplots with the enrichplot package v1.14.1. All other figures were created using the ggplot2 package v3.3.5 [36]. To combine individual figures the multipanelfigure package v2.1.2 was used [37]. To test for significant overlap between DEGs and DMRs, hypergeometric tests were performed using the base R function phyper. Statistical analyses of pyrosequencing and qPCR were done by GraphPad Prism software using the above-described statistical tests. Statistical significance was denoted as following: *p* value = ns; non-significant, * *p* < 0.05, ** *p* < 0.01 and *** *p* < 0.001.

## 3. Results

Histological assessment of bladder specimens after chronic exposure to BBN (Figure 1A) revealed urothelial cancers of diverse tissue architecture, cell morphology and tumor stage progression (Figure 1B). The specimens either showed invasive tumors (*n* = 11), identified as an abnormal mass of cancer cells in the subepithelial connective tissue with or without muscle invasion, or tumors without invasion beyond basal membrane of the epithelium (*n* = 6), with four non-invasive tumors displaying characteristics of carcinoma in situ, while two did not show signs of cancer pathology. Non-treated, age-matched controls showed regular tissue morphology. Samples were thenceforth stratified into invasive (I), non-invasive (NI) and control (CTRL) groups for further methylome and transcriptome profiling with the aim of identifying the molecular changes that are correlated with the progression to invasive tumor after long term BBN exposure.

**Figure 2:**
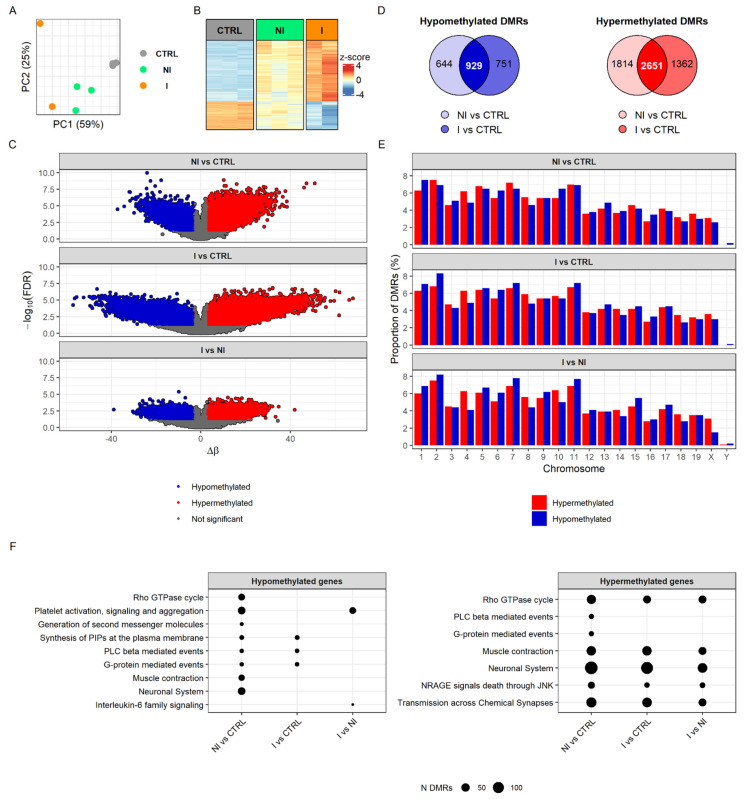
DNA methylation analysis of mouse bladder cancers by Infinium™ Mouse Methylation BeadChip. (**A**) Principal components analysis (PCA) score plot of the full methylome. (**B**) Heatmap showing overlapping of significant differentially methylated probes (DMPs) of the three different comparisons (NI vs. CTRL, I vs. CTRL and I vs. NI; n DMPs = 6291) with the cut off |delta beta values| > 0.25. (**C**) Volcano plots summarizing methylome analysis of a different distribution of hyper- and hypomethylated DMPs expressed with delta β and *p*-value in each comparison. (**D**) Venn diagrams of the overlap of hyper- and hypomethylated differentially methylated regions (DMRs) identified in non-invasive and invasive groups relative to controls with applied cut off |mean methylation difference| > 3. (**E**) The proportion of DMRs distributed along the chromosomes. (**F**) Top Reactome pathways enriched in hypo- or hypermethylated DMRs in each comparison. I, invasive; NI, non-invasive; CTRL, control.

Several reports indicated that methylation changes could be detected in the early phase of bladder tumor development [15,21,38]. To characterize DNA methylation changes associated with BC progression, we profiled the DNA methylome of BBN-induced non-invasive and invasive bladder tumors and control (i.e., untreated) samples using an Infinium™ Mouse Methylation BeadChip (Illumina Inc., San Diego, CA, USA), which allowed for the robust interrogation of >285,000 methylation sites per sample at single-nucleotide resolution. Principal component analysis (PCA) of differentially methylated regions (DMRs) separated all three sample groups, invasive from non-invasive tumors and both tumor groups from control samples (Figure 2A). Methylome profiling demonstrated consistency and conserved patterns in the heatmap of DMRs from the replicates of invasive and non-invasive tumors as well as control samples (Figure 2B). The heatmap of DMRs displayed, unambiguously, different DNA methylation patterns between controls and tumors, with relatively more distinct difference in invasive tumors (Figure 2B,C).

Of the 1680 hypomethylated DMRs found when comparing the invasion group to control, 929 were common to the non-invasive group versus control DMRs. In the case of the 4013 hypermethylated DMRs in the invasion group, 2651 were shared with the non-invasion group (Figure 2D). Examining the genomic methylation pattern indicated that extensive epigenetic alterations accompany cell transformation. However, when invasive BCs were compared with the non-invasive BCs, marked hypomethylation changes were detected among most of the chromosomes, indicating DNA demethylation as an important element in urothelial cancer progression from non-invasive into invasive BC (Figure 2E). For a comprehensive overview, the biological functions of DMRs were determined based on Reactome pathways. Hypomethylation of non-invasive tumors (compared to controls) was associated with platelet activation, signaling and aggregation, Rho GTPase cycle, neuronal system and muscle contractions, whereas hypomethylation in the invasive tumors’ group (when compared to controls) affected pathways for phosphatidylinositol phosphates (PIPs) synthesis and the phospholipase C (PLC) beta and G-protein-mediated events, although these pathways were present in the non-invasive group as well. The pattern of DMRs hypermethylation found in non-invasive and invasive tumors was associated with muscle contraction, the neuronal system, transmission across chemical synapses, Rho GTPase cycle and NRAGE signals death through JUN Kinase (JNK) (Figure 2F). Overall, we observed that common pathways were affected more by hypermethylation in both invasive and non-invasive groups then hypomethylation. Yet, interestingly, hypo- and hypermethylation had an impact on muscle contraction and neuronal system related processes in non-invasive tumors, while, in the invasive tumors, we found only hypermethylated DMRs related to these biological pathways. Direct comparison of hypomethylation profiles between non-invasive and invasive BCs revealed a difference in platelet activation and in IL-6 signaling, while hypermethylation profile was similar between groups. The full results of DNA methylation analysis can be found in Supplementary Table S3.

**Figure 3:**
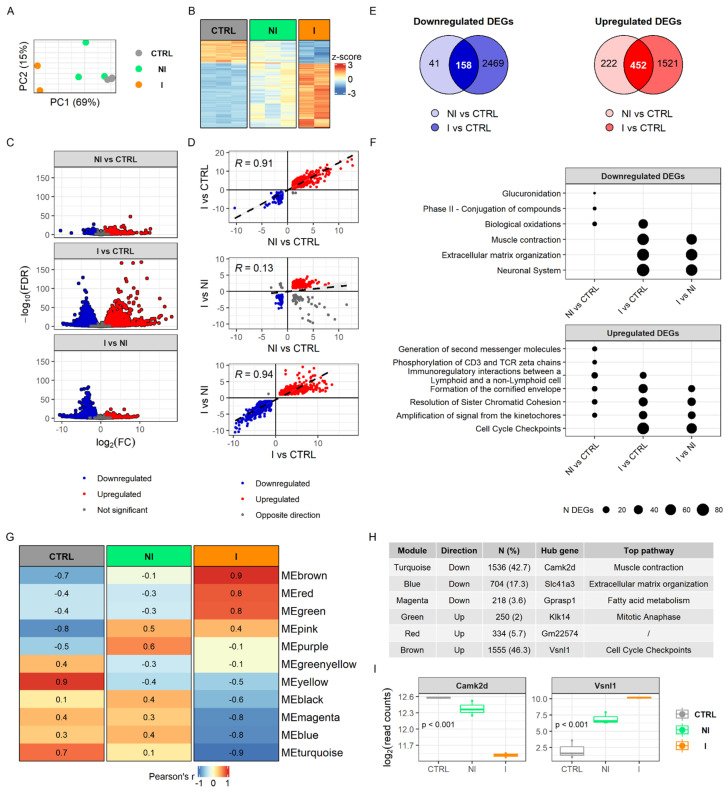
Gene expression profiling of mouse bladder cancers. (**A**) Principal component analysis (PCA) score plot showing sample grouping. (**B**) Heatmap of significant differentially expressed genes (DEGs) in overlap between three comparisons (NI vs. CTRL, I vs. CTRL, and I vs. NI; n DEGs = 234). (**C**) Volcano plots with log_2_ fold change and FDR adjusted *p*-value in each comparison. (**D**) Correlation between log_2_ fold change of DEGs across three comparisons with Pearson’s R correlation coefficient. (**E**) Venn diagrams of the overlap of up- and downregulated DEGs identified in non-invasive and invasive groups relative to their controls. (**F**) Top Reactome pathways enriched in down- and upregulated DEGs. (**G**) Heatmap of module eigengenes and Pearson correlation with phenotype, results from weighted gene co-expression network analysis (WGCNA), values higher than 0.75 show statistically significant correlations. (**H**) Summary of central module genes (N-number of genes; percentage represents a portion of these genes identified as DEGs) and top hub genes from WGCNA analysis of modules significantly correlated with invasion, with the most prominent Reactome pathway associated with a particular module. (**I**) Boxplots of normalized read counts from two hub genes representing the most significantly down- and upregulated modules. I, invasive; NI, non-invasive; CTRL, control.

To determine how the detected methylome changes ultimately affect the transcriptome, we performed RNA sequencing (RNA-seq) on the same sample set. The results showed that the transcriptome of the urinary bladder is markedly affected by chronic BBN treatment and is dependent on tumor invasiveness. Principal component analysis of gene expression profiles showed a clear grouping of samples, while the non-invasive tumor samples revealed heterogeneity (Figure 3A), which is consistent with histopathological differences of tumor pathology (Figure 1B). The heatmap plotted with scaled normalized counts of 234 shared differentially expressed genes (DEGs) between three comparisons displayed non-invasion as an intermediate stage in between the control and the invasive group (Figure 3B). Among the samples, two from the non-invasive group share similar patterns with controls while the third showed an expression profile similar to invasive tumors but with less pronounced change in expression. In general, invasive bladder tumors were characterized by more downregulated DEGs than non-invasive ones when both were compared with the control group (Figure 3C). This is also visible from correlation analysis of DEGs showing lack of correlation when comparing non-invasion groups with control DEGs with invasion versus non-invasion DEGs (R = 0.13, *p* = 0.025) (Figure 3D). Furthermore, by overlapping DEGs after comparing tumor tissues relative to control, we found that non-invasion and invasion share more upregulated DEGs than downregulated genes (*n* = 452, *n* = 158 respectively). In invasive bladder tumors we detected noticeably higher RNA expression alterations, including more DEGs as well as higher average fold changes and more significant *p*-values, in comparison with a non-invasive group (Figure 3C,E).

Functional enrichment analysis of DEGs highlighted muscle contraction, extracellular matrix organization and the neuronal system as downregulated processes within the invasive group, but not in the non-invasive (Figure 3F). Since muscle contraction and neuronal system had apparent changes in the methylome in BC groups, transcriptome changes likely become evident when a tumor develops invasive traits. Furthermore, detoxification processes of phase II conjugation, especially glucuronidation, were downregulated only in the non-invasive tumor samples. Analysis of enriched pathways from the upregulated DEGs revealed molecular pathways involved in immunoregulation and mitosis. Cell cycle checkpoints were upregulated only in the invasive group, while T-cell signaling was upregulated exclusively in the non-invasive group. The full results of RNA-seq analysis can be found in Supplementary Table S4.

Enrichment analysis of modules derived by the unsupervised weighted network correlation approach (WGCNA) revealed similar findings to the enrichment analysis of DEGs. We identified 11 clusters of co-expressed genes and calculated eigengenes to summarize the expression profiles within modules (Figure 3G,H). Interestingly, only the invasive group showed significantly correlated modules, Pearson’s R > 0.7, (six in total, of which three were positively and three negatively correlated with invasion), and the pattern was quite different from the non-invasive group. Each module of co-expressed genes contained a set of genes that are considered to be involved in the same biological pathway. Two of the most prominently downregulated modules in invasive tumors were associated with pathways of muscle contraction and extracellular matrix organization by over-representation analysis, using the Reactome Pathways database. Genes clustered in the upregulated modules are part of cell cycle checkpoints and mitotic anaphase pathways. Hub gene Camk2d was the most significantly changed gene within a downregulated module (i.e., muscle contraction) and Vsnl1 gene, related to cell cycle checkpoints, is highly expressed in the invasive tumor group (Figure 3I). The full results of WGCNA analysis can be found in Supplementary Table S4.

**Figure 4:**
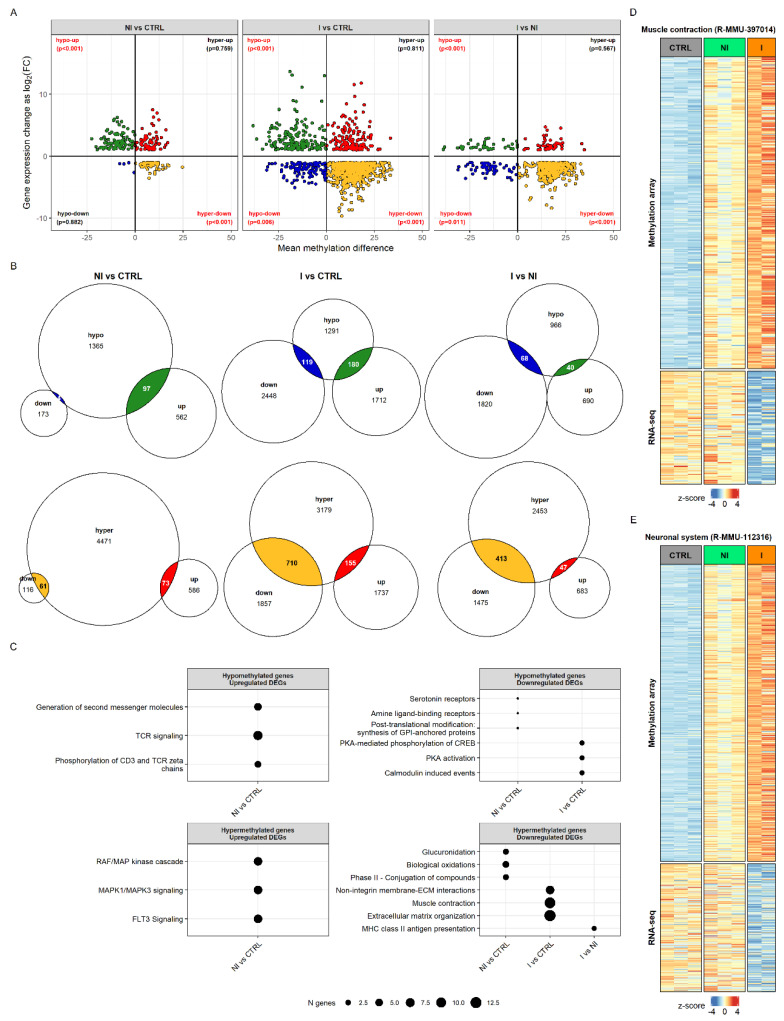
The relationship between DNA methylation and gene expression in BBN-induced bladder tumors in mice. (**A**) Scatter plot of mean methylation difference versus log_2_ expression change by applying cut off |mean methylation difference| > 3 and |log_2_ fold change| > 1, FDR adjusted *p*-value < 0.05; *p*-values obtained with hypergeometric tests. (**B**) Venn diagrams summarizing the intersection between differentially expressed genes (DEGs) and differentially methylated regions (DMRs). (**C**) Top Reactome pathways enriched in different comparisons (NI vs. CTRL, I vs. CTRL, and I vs. NI) based on the intersection between DEGs and DMRs. (**D**) Clustering of samples according to DNA methylation and gene expression levels of genes annotated to Reactome muscle contraction and (**E**) neuronal system pathways. I, invasive; NI, non-invasive; CTRL, control.

To determine whether the observed differences in transcriptome can be explained by the changes in the genes’ methylation status, we compared the results of the DNA methylation and RNA-seq analyses. Hypermethylation status was positively correlated with downregulation in its expression by hypergeometric test (*p*-values shown on Figure 4A). The overlap of significant genes obtained by the two analyses was the following: 28.11% of DEGs and 3.87% of DMRs were in overlap for NI vs. CTRL, 26.10% of DEGs and 20.66% of DMRs for I vs. CTRL, and 26.20% of DEGs and 14.24% of DMRs for I vs. NI (Figure 4B).

To assess the biological significance of gene sets that shared methylation and transcriptional status per each sample group, we performed gene over-representation analysis using Reactome pathways (Figure 4C). Hypomethylation corresponded to upregulation in the case of genes involved in T-cell signaling but only in the non-invasive group. Hypermethylated and upregulated genes in the non-invasive group were associated with MAPK and FLT3 signaling pathways. In the invasive group the hypomethylated and downregulated statuses of genes were associated with protein kinase A (PKA) and calmodulin induced events. The largest portion of significant genes was hypermethylated and had a lower gene expression than did control for both tumor groups. For the non-invasive group, this included genes involved in glucuronidation and biological oxidations, while, for the invasive group, genes were found that affect muscle contraction, extracellular matrix organization and non-integrin membrane-ECM interactions.

Interestingly, even though the muscle contraction and neuronal system genes (Reactome pathways R-MMU-397014 and R-MMU-112316) did not show differences in expression level in the non-invasive group, their methylation status shows hypermethylation, as in the invasive group, but with smaller changes in the percentage of CpG methylation. Non-invasive samples clearly group with controls in muscle genes’ transcriptional status, whereas, in the methylation pattern, they appear already similar to the invasive group (Figure 4D). The same pattern was observed when taking into account genes annotated to the neuronal system (Figure 4E). The full results of functional enrichment analysis in overlap between DNA methylome and transcriptome can be found in Supplementary Table S5.

**Figure 5:**
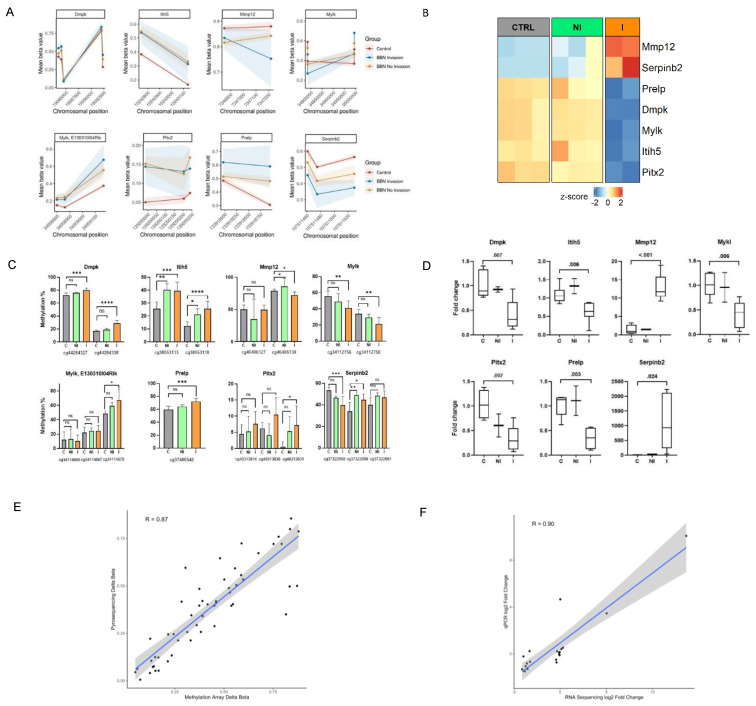
Validation of the changes in expression and methylation at selected gene targets. Comparison of DNA methylation mean beta value measured by Methylation BeadChip Array (**A**) and RNA-seq expression (**B**) patterns (scaled normalized counts) of particular gene targets in BBN-treated mice with regard to their controls. (**C**) DNA methylation levels of the CpG sites (indicated below x axis) showing mean methylation in validation samples. The number of mice in the control group was 8, three in the non-invasive group and nine in the invasive BC group. Statistical analysis was performed using Kruskal–Wallis ANOVA with multiple comparisons. (**D**) Validation of gene expression was done on five control mice, two non-invasive samples and six invasive tumors. Values were normalized to Actb and presented as a fold change. Statistical analysis was performed using unpaired T-test between C and I. Pearson correlation of (**E**) methylation levels between pyrosequencing and Infinium™ Mouse Methylation BeadChip, and (**F**) gene expression amongst qPCR and RNA-seq. Dots indicate individual samples. C, control; NI, non-invasion; I, invasion. * *p* < 0.05; ** *p* < 0.01; *** *p* < 0.001; **** *p* < 0.0001; ns, not significant.

Finally, to validate the methylation and transcriptional changes identified by genome-wide approaches we conducted targeted pyrosequencing and qPCR analyses, respectively, on a large number of collected BC tumors and control specimens different from the one previously subjected to the genome-wide analyses (Figure 1A). Validation genes were selected by overlapping the methylation profiling with RNA-seq results to find potentially interesting targets which could contribute to invasive traits in muscle-invasive BC. Genes were chosen based on their significance in both analyses, with an FDR-adjusted *p*-value < 0.05 and with |delta β-values | > 0.25 for DMRs and ± two-fold change for DEGs. (Figure 5A,B). The selected genes are mainly involved in the muscle and extracellular matrix processes, which were shown by functional enrichment analysis of methylome and transcriptome to represent important pathways distinguishing the invasive and non-invasive groups. We confirmed the presence of methylation changes in DMRs, as well as changes in expression level for the invasive group (Figure 5C,D). Hypermethylation over the DMR of Itih5 gene was associated with a reduced gene expression in the invasive group, while in the non-invasive group it remained similar to the expression level observed in controls. Similarly, increased methylation levels in one CpG site of Mmp12 (probe ID: cg46406130) could be partly responsible for the observed upregulation in this gene. In addition, within the invasive samples, Serpinb2 (cg37322058 and cg37322059) displayed hypo- and hypermethylation, respectively, on two neighboring CpG sites, which could have resulted in the increased amount of its transcripts. A possible explanation could be that CpG (corresponding to cg37322058 probe) is nearest to the promoter region of the gene.

Furthermore, the results of the validation assays showed high correlation with the sequencing/array results (Figure 5E,F). In the non-invasive group methylation changes were not always statistically significant, partly because of the small number of available additional samples for validation; however, their methylation status was altered, while mRNA expression did not change, further confirming the results from RNA-seq and Methylation BeadChip Array.

## 4. Discussion

Muscle invasive urinary bladder cancer remains a disease with poor prognosis accompanied by high progression rate to metastatic disease and substantial mortality rate [1]. Several studies of the mutational landscape of urothelial bladder tumors identified the high frequency of mutations in genes involved in epigenetic regulation, suggesting that the deregulation of epigenetic mechanisms has an important role in bladder carcinogenesis [39]. Since DNA methylation changes—a major epigenetic mechanism implicated in tumorigenesis—precede modulations in gene expression [40] we reasoned they could be used to predict tumor invasiveness. In this study, we aimed to identify changes across the methylome and transcriptome of non-invasive and invasive bladder cancers using the mouse model of BBN-induced bladder cancer. Methylome profiling revealed graduality of methylation between non-invasive and invasive tumors, which suggests the existence of a tumor methylation pattern that increasingly changes as the tumor progresses. We observed CpGs of genes involved in muscle contraction and neuronal system pathways to be among the ones mostly affected by hypo- and hypermethylation in non-invasive tumors, whereas the invasive tumors showed only hypermethylation in the same biological processes. This could be a reflection of the cell heterogeneity of non-invasive tumors, as well as the domination of one cell type as the tumor becomes invasive.

On the transcriptome level, muscle pathways were downregulated in the invasive group, but not in the non-invasive group, indicating the extent of the methylation changes detected in non-invasive tumors was not sufficient to change gene expression. Thus, methylation changes in these sets of genes are present in earlier stages of the disease and further augmented throughout tumor progression. These findings are consistent with the study by Gao et al. [41] on human samples, which identified the downregulation of genes involved in vascular smooth muscle contraction and the extracellular matrix. Additionally, in a study by Han et al. [42], the authors identified muscle contraction and muscle system processes as the most downregulated processes in bladder cancer, which is in concordance with our findings. Altogether, this emphasizes the importance of deregulation of muscle processes early in bladder carcinogenesis.

Besides muscle contraction, we identified changes in neuronal system genes with the same pattern, i.e., methylomes were affected in non-invasive and invasive tumors, while transcriptomes only in the latter. Neuronal changes could reflect deregulation of urothelial–neuronal interactions due to exposure to BBN. Urothelial cells exhibit both sensor and transducer properties that are altered in different pathological states, resulting in modification of local sensory nerves that control muscle contraction [43]. The urinary bladder smooth muscle is excitable by multiple ion channels, primarily calcium channels like Cav1, Cacna1c, Atp2b4, and transient receptor potential (Trp) channels [43], many of which showed, in our study, changes in expression and methylation pattern in invasive tumors. Changes in bladder contractility is a known complication of BC, and alterations in detrusor contractility was found in the rat model of bladder cancer, confirming our findings and stressing the importance of neuro-muscle disturbances in BC [44].

With a specific focus on genes predominantly changed in invasive carcinoma, we selected gene targets to validate our results on more tumor samples. The validation genes were chosen mainly for their involvement in the muscle contraction processes. Among the genes validated, Itih5 (probe ID: cg38053113 and cg38053118), Mmp12 (cg46406130) and Serpinb2 (cg37322059) showed significant methylation changes of particular CpGs across BC tumors. Among the validated DMRs, we observed gradual changes in CpG methylation from non-invasive to invasive tumor group which were even observable after acute, two weeks, BBN exposure (Supplementary Figure S2). This observation suggests that DNA methylation is happening during the earliest phases of BC development and needs to be followed more deeply in future studies. Expression of the same genes showed significant changes in the invasive tumors, while the non-invasive group had similar expression as controls, confirming results from our RNA-seq and DNA methylation array analyses.

The biological significance of the selected genes in BC could be explained by previous observations, including their high mutational rate in human BC, according to the cBioPortal database [45,46] (Supplementary Figure S3). Namely, Inter-α-trypsin inhibitor heavy chain 5 (*ITIH5*) is epigenetically altered in various cancers, including BC. Its promoter hypermethylation was related to progressive types of BC [47,48]. Furthermore, *ITIH5* has a role in the extracellular matrix stabilization and the prevention of metastasis. Its disruption could influence focal adhesion—one of the KEGG pathways whose methylation was deregulated in non-invasive and invasive tumor stages (Supplementary Figure S4). Matrix metalloproteinases 12 (*MMP12*) has been associated with tumor cancer progression with its implications in modulation of tumor environment through degradation of extracellular matrix. Previous findings emphasized the role of this protein family in BC [49,50,51]. To our knowledge, alterations in the DNA methylation of *MMP12* were not reported previously, so this could be taken into account in future studies. Finally, currently there is poor evidence to support the role of *SERPINB2* in BC development but its enhanced expression has been linked with metastatic progression in various cancer types [52].

Furthermore, other biological processes are affected by malignant transformation of the urothelium, namely xenobiotic phase II metabolism (i.e., glucuronidation), T-cell signaling, different signaling cascades (MAP kinase cascades, FLT3 signaling) and biological oxidations indicating profound changes in the tissue during the course of malignant transformation of the urinary bladder.

In summary, we identified potential molecular targets that contribute to the pathogenesis of muscle-invasive bladder cancer. These genes could potentially serve as biomarkers in the development of epigenetic-based strategies for monitoring patients with bladder cancer and reducing BC progression potential and its recurrence rates. Furthermore, the results of this study can advance our understanding of the underlying biology of BC progression. Transcriptional shifts of muscle and neuronal genes are correlated with the invasiveness of bladder cancer, while their epigenetic deregulation occurs earlier in the process of carcinogenesis, with potential for predicting tumor invasion. Changes in mice tumors duplicate human bladder cancer attributes, indicating that novel findings from our study could be clinically relevant.

## 5. Conclusions

DNA methylome and gene expression profiles exhibit extensive changes during urinary bladder carcinogenesis. Methylation and gene expression run in opposite directions suggesting their mechanistic interconnection. Methylation changes in the genes involved in muscle- and neuronal-related processes were found in non-invasive bladder cancers and were more pronounced in invasive BCs. Our findings argue that early epigenetic changes in muscle and neuronal processes are revealing mechanisms of bladder carcinogenesis that might be a basis for its invasiveness, which could be exploited in early diagnosis programs as well as in novel therapeutic approaches.

## Data Availability

Processed data of RNA-seq and Methylation Array are available at Supplementary Materials.

## Supplementary Materials

The following supporting information can be downloaded at: https://www.mdpi.com/article/10.3390/cancers14030487/s1, Figure S1: Quality control of Infinium Mouse Methylation BeadChip data; Table S1: Primer sequences and PCR conditions used for pyrosequencing; Table S2: List of primers used for qPCR analysis. Figure S2: Short treatment of BBN exposure. Figure S3: Somatic mutation and alteration frequencies analysis of gene targets across different cancer types in cBioPortal database (TCGA PanCancer Atlas Studies). Figure S4: The overlap of DMRs between invasive and non-invasive groups. Table S3: Full results of DNA methylation analysis. Table S4: Full results of RNA-seq analysis. Table S5: Full results of functional enrichment analysis in overlap between DNA methylome and transcriptome.
