# Supplementary material for: DNA Methylome Changes of Muscle- and Neuronal-Related Processes Precede Bladder Cancer Invasiveness
Source: Cancers (Basel). 2022 Jan 19;14(3):487. doi: 10.3390/cancers14030487 (PMC8833512; doi:10.3390/cancers14030487)
Supplement: Supplementary file 1 [file cancers-14-00487-s001.zip › cancers-1545106-supplementary/Supplement_DNA methylome changes of muscle and neuronal related processes precede bladder cancer invasiveness FV/Supplment Figure S1-S4; Table S1-S2.pdf]

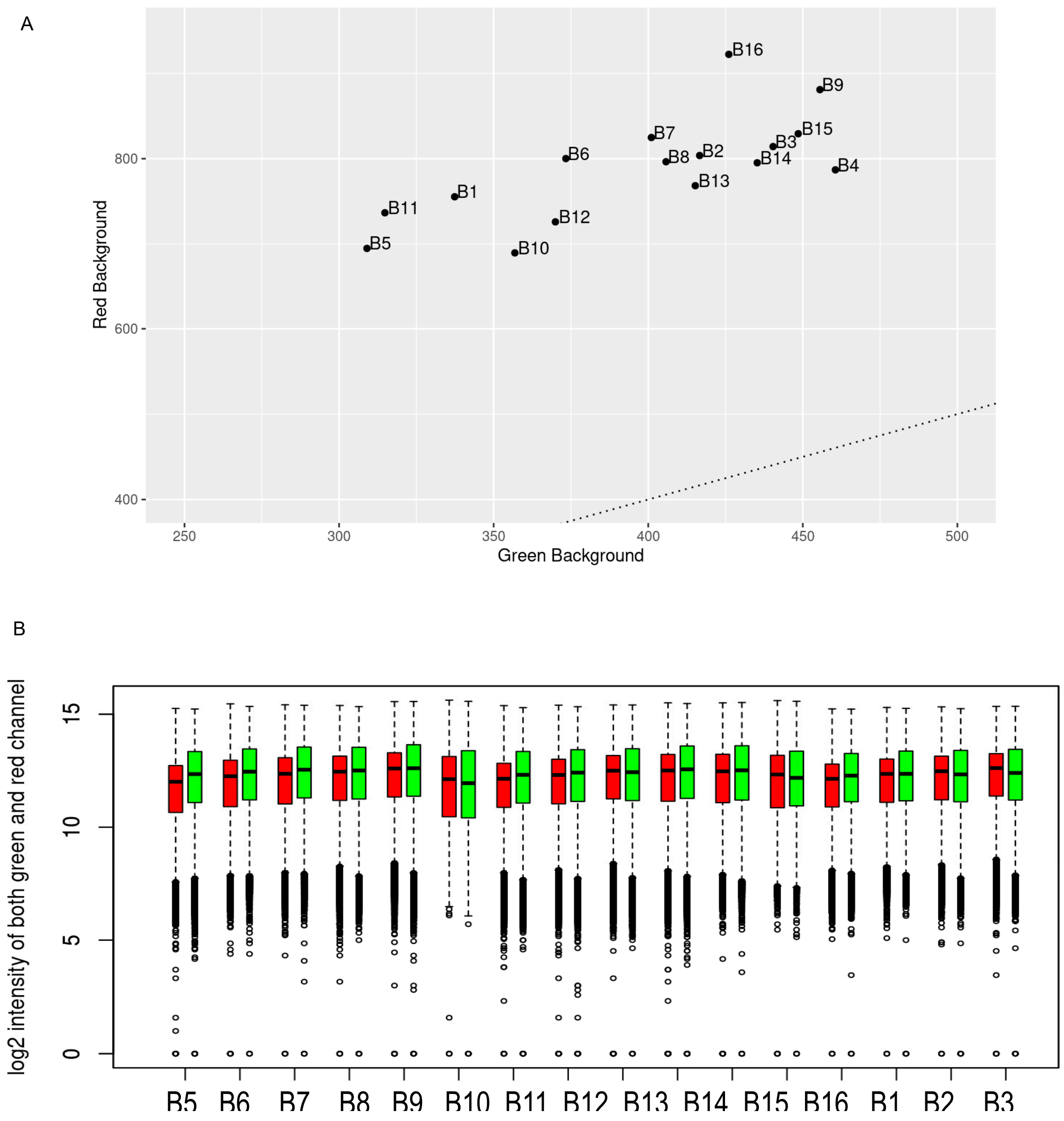


**Supplementary Figure S1.** Quality control of Infinium Mouse Methylation BeadChip data. **(A)** QC plot of $log_2$ median intensities in red and green detection channels. The cut-off for acceptable sample quality is denoted by the dashed diagonal line and demarcates the points where the average of red and green channel $log_2$ median intensities is 10.5. **(B)** Boxplots showing the spread of $log_2$ intensities in both red and green across analyzed samples.

**Supplementary Table S1.** Primer sequences and PCR conditions used for pyrosequencing.

| Gene locus | Forward Primer (5'-3') | Reverse Primer (5'-3')[1] | Sequencing Primer (5'-3') | PCR conditions[2] | |
|---|---|---|---|---|---|
| | | | | **A** | **X** |
| **Dmpk** | TGGAGATGTTGTAGGTTTTAGG | b-CAACTTTCTTCTAAAACCCCCA | TGTTGTAGGTTTTAGGAGT<br>TATTTATAGTTTTTTTTT | 60°C | 50 |
| **Itih5** | GGAGATGATTTAGTTTGGTAGG | b-TACTCCTAACCCACAACTAATC | TTTAGTTTGGTAGGAGTGTT<br>ATATTGTTAGGGATATTT | 60°C | 50 |
| **Mmp12** | TGGTGGGATTAAGTGAGTGTTGAA | b-TCATCACTCATCTCTATACCTCTC | AGTATGAAAGATGAGGAA<br>GGTGGAAGTTAAGATTTAT | 59°C | 55 |
| **Mylk, Rik** | TGYGGGAGTTGTAGTTTTGA | b-TATCCCTCACTTAAACCAACTT | TTTTTTATTTTTTATTGT<br>GGAGTAATATGTTAAATGT<br>GTTTTTAAGTATTTGAAA | 58°C | 55 |
| **Mylk** | GGTGAAGAAGGTTTTTTTTGGAATTG | b-CACCCCTCCACCCTTTCA | TTTAGAATATTTTTTGGTA<br>AGAGTTGTAGAGGGGAATT | 59°C | 50 |
| **Pitx2** | GAAGAGAGAGGGATGAGAG | b-ACTACCAACCATACCTAACTTC | AGTAGTAGATAGAAATTGT<br>TAGAGTTGTGAGATAATG<br>GTTTTATTTTGGAGAATTTT | 58°C | 50 |
| **Prelp** | ATGAATTGGGGTTGGGTAAAGT | b-ATCTAATTTCTTCCCCATTCCC | GTAGAGTAAATATTATATTT | 60°C | 50 |
| **Serpinb2** | TGTGTGGGAGGGGTAGA | b-CTTACCCTCTACACAACTCA | GTATTTTTTGTGTGTTAGT<br>TTTTTAGTGAATTTAGGG | 56°C | 50 |

[1]**b** represents biotin

[2]PCR conditions: 94°C for 15 min (1 cycle), 94°C for 30 s, **A**°C for 30 s, 72°C for 30 s (**X** cycles), 72°C for 5 min (1 cycle)

**Supplementary Table S2.** List of primers used for qPCR analysis.

| Gene target | Primer (5'-3') |
|---|---|
| Dmpk_F | CCCCTTTACACCAGACTTCG |
| Dmpk_R | ATGTCTTCCTGCATGTCTGAC |
| Itih5_F | TGCTTTAACATCGACGGAGAG |
| Itih5_R | GGTGATGGTGCGGAAGTAG |
| Mmp12_F | CTTAACCCCAGCACATTTCG |
| Mmp12_R | GTGACAGCATCAAAACTCAAGC |
| Mylk_F | TTGTGGCTCCTGAAGTGATC |
| Mylk_R | TGAAGTGACGTTGGCTAAGG |
| Pitx2_F | CAGAGGACTCATTTCACTAGCC |
| Pitx2_R | CGGCGATTCTTGAACCAAAC |
| Prelp_F | GGGAGGAACAGAAGAGTGC |
| Prelp_R | GTTTTGGTCTTGTCGGTTGG |
| Serpinb2_F | CCCGAAGGTTCTGTAGATGAAG |
| Serpinb2_R | CTGGACAGGTATGCTCTCATG |
| Actb_F | CACTGTCGAGTCGCGTCC |
| Actb_R | TCATCCATGGCGAACTGGTG |

* F indicate forward primer and R reverse.

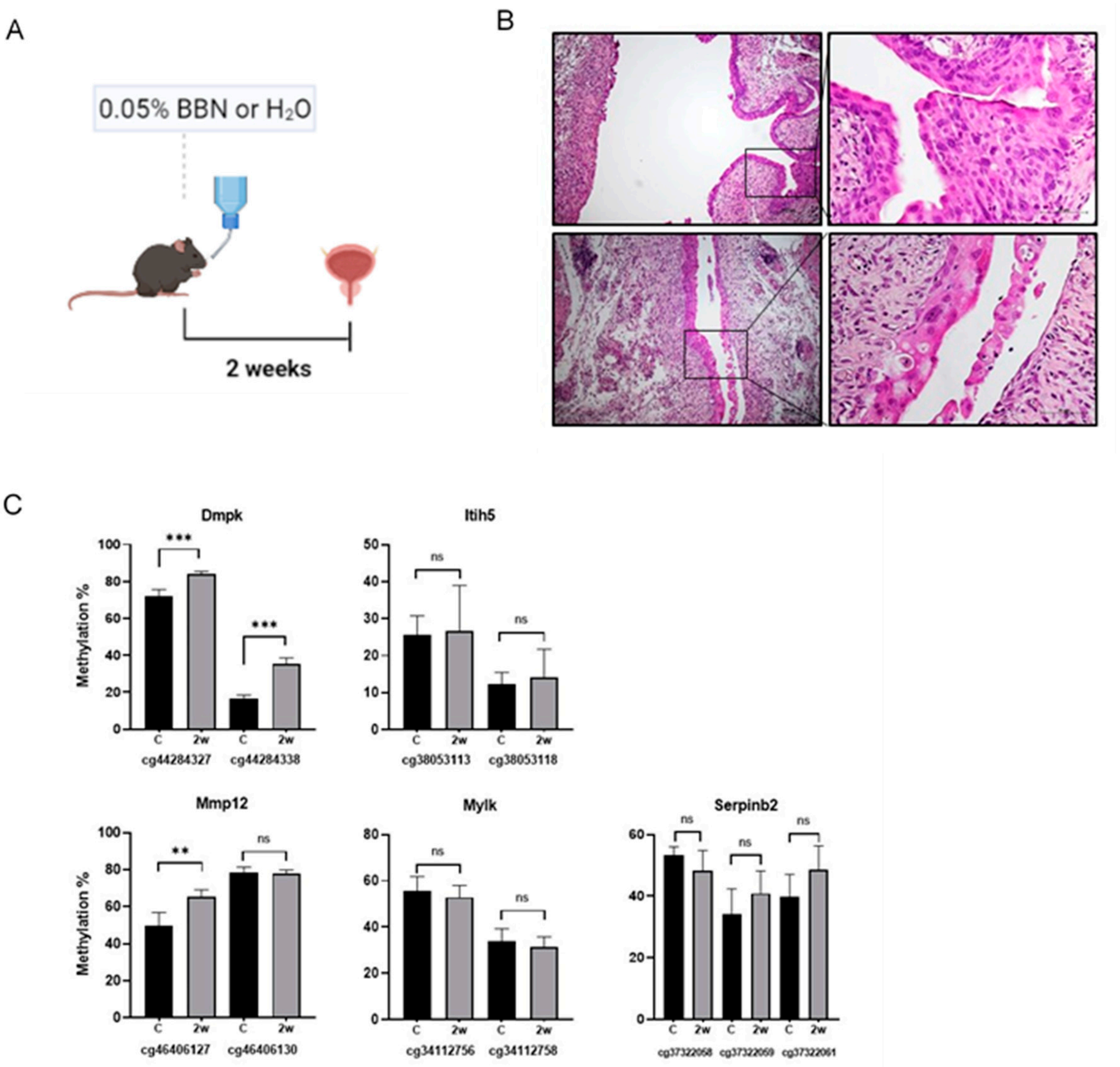


**Supplementary Figure S2.** Short treatment of BBN exposure. (**A**) A schematic representation of short BBN treatment and paired controls (Created with BioRender.com). (**B**) Histopathological analysis of bladder tissue after 2 weeks of BBN exposure characterized by reactive atypia and influx of inflammatory cells. (**C**) DNA methylation levels of the CpG sites (indicated below x axis) showing mean methylation in validation samples. Number of mice in the control group was 8 and in 2 weeks BBN-treated was 4. Statistical analysis was performed using Unpaired T-test. *$p < 0.05$; ** $p < 0.01$; *** $p < 0.001$; **** $p < 0.0001$; ns, not significant. C, control; 2w, 2 weeks of BBN-exposure.

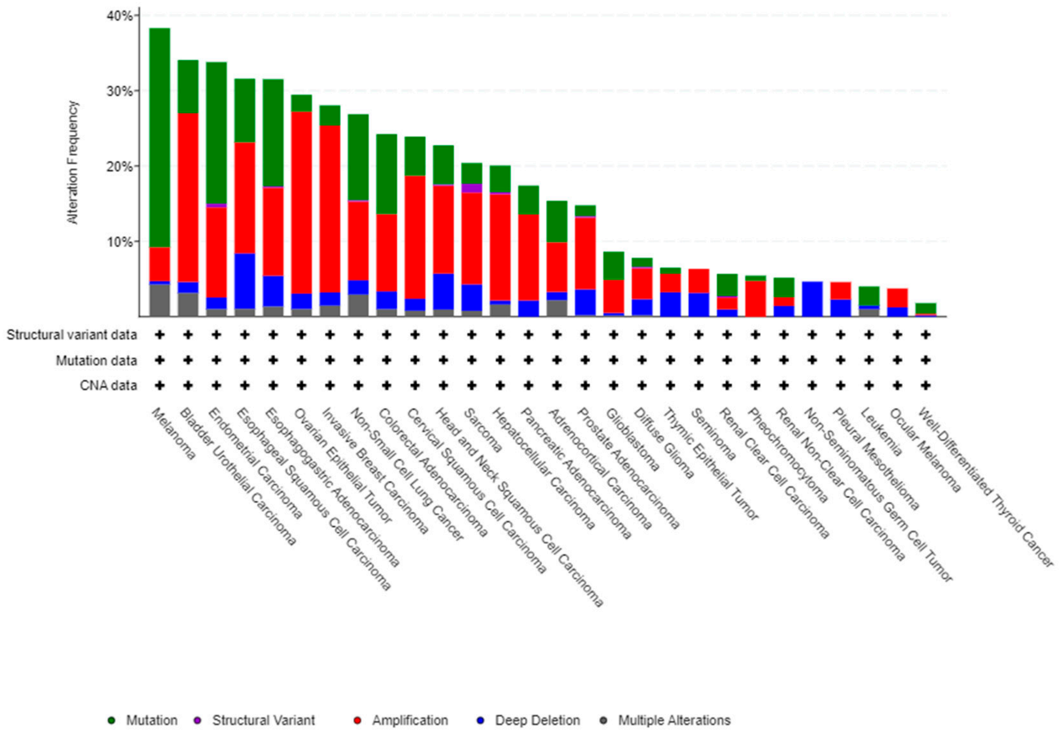


**Supplementary Figure S3.** Mutational status of validated genes across different cancer types. Somatic mutation and alteration frequencies analysis of *DMPK*, *ITIH5*, *MMP12*, *MYLK*, *PRELP*, *PITX2* and *SERPINB2* in cBioPortal database (TCGA PanCancer Atlas Studies).

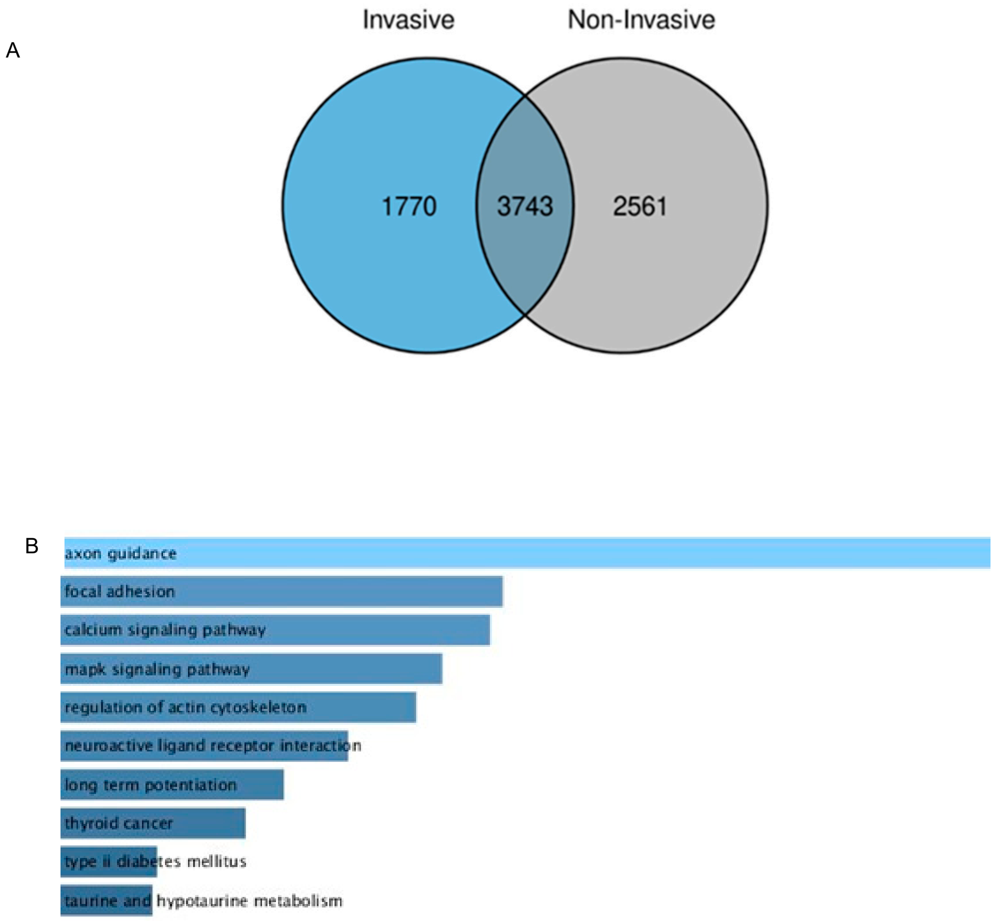


**Supplementary Figure S4**. The overlap between invasive and non-invasive groups. (**A**) Venn diagram showing the overlap of the numbers of differentially methylated regions (DMRs) detected between invasive and non-invasive groups compared to controls; (**B**) KEGG pathway performed on 3743 DMRs from the overlapped region. All pathways in blue show $p < 0.05$.
